# Affects of ableism in medical education: happiness, resignation, and the disabled killjoy

**DOI:** 10.1007/s10459-025-10499-4

**Published:** 2026-02-02

**Authors:** Erene Stergiopoulos, Neera R. Jain

**Affiliations:** 1 The Wilson Centre, Institute for Health Policy, Management, and Evaluation, University of Toronto, Toronto, Canada; 2 Department of Psychiatry, Temerty Faculty of Medicine, University of Toronto, Toronto, Canada; 3 Centre for Medical and Health Sciences Education, School of Medicine, Faculty of Medical and Health Sciences, Waipapa Taumata Rau - The University of Auckland, Auckland, New Zealand

**Keywords:** Disability, Ableism, Medical students, Affect theory, Emotions, Critical disability studies

## Abstract

Exclusion is not only an experience but a feeling. Yet, studies of disabled medical learners have paid little attention to their emotions. Affect theory provides a lens for studying how emotions are produced by socio-political ways of being and knowing. In this study, we investigated affects circulating through disabled student narratives to explore how these students perceived disability and agency within an ableist system. We conducted a reflexive thematic analysis of 674 open-text responses by disabled medical students from the 2019 and 2020 AAMC Year Two Questionnaire about their experiences with disability in medical school. We interpreted themes using affect theory and critical disability theory to map the interconnections between students’ emotions, the political actions they took, and where they responsibilized disability and change. Three primary affects circulated in these texts. Happiness underpinned students’ expressions of gratitude, awe, and institutional compliance with any accommodations they received, for disabilities they considered their own responsibility. Frustration accompanied descriptions of institutional silencing or failure to accommodate, where students emphasized environmental learning barriers, system failures, and embodied the “disabled killjoy” who resisted system constraints. Resignation circulated among students who gave up on institutional access, and resorted to managing disability alone in a system they assumed would not change. These students internalized principles of self-regulation to manage their disability, providing a novel perspective on disability non-disclosure in medical education. Affect theory provides an underexplored lens for studying the relations between emotions power, and highlights the need for nuance in interpreting disabled students’ emotions.

## Introduction

*“The moment when a feeling enters the body is political.” — Adrienne Rich*, “The Blue Ghazals,” in *The Will to Change* (1971).

Exclusion is a visceral feeling. Yet research on disabled medical learners, who are systematically excluded from training, has paid little attention to their emotions. While the growing field of disability in medical education has studied the forms and consequences of ableism (Battalova et al. 2020; Brown and Finn 2024; Jain 2022, 2024; Jain et al. 2024; Jarus et al. 2023a, b; Shaw et al. 2023; Stergiopoulos et al. 2018; Stergiopoulos and Martimianakis 2023), we have not yet investigated the *emotions* that arise from, underpin, and reproduce this injustice. In this paper, we foreground disabled learners’ emotions, to understand how emotions are produced by particular socio-political ways of being and knowing in an ableist system.

Ableism is, according to Campbell (2009, p. 44), “a network of beliefs, processes and practices that produce a particular kind of self and body (the corporeal standard) that is projected as perfect, species-typical and therefore essential and fully human.” While ableism comprises a number of practices, within medical education ableism is operationalized through policies, language, and practices that explicitly and implicitly demarcate which learners belong and which ones do not, according to ability (Bulk et al. 2017; Jarus et al. 2023b; McKee et al. 2020; Stauffer et al. 2022; Stergiopoulos et al. 2018; Zazove et al. 2016). This standard of ability reflects what Jain (2022) terms the *capability imperative*, a cultural logic that assumes and requires hyper-ablebodiedness and mindedness from physicians. The capability imperative becomes a taken-for-granted assumption for learners and teachers, outlining a normative way of being a ‘good doctor’, and justifying and perpetuating an ableist status quo. The result is a medical training environment in which ableism is “intrinsic, invisible, and unquestioned,” (Watermeyer 2025, p. 123).

Disability is increasingly claimed and recognized in medical training, where it has influenced a growing wave of research over the last decade (British Medical Association 2020; Meeks et al. 2019; Meeks et al. 2022a, b). In a large national 2024 survey, 17.6% of U.S. medical students anonymously self-reported disability, with 43.4% stating they disclosed their disability and sought accommodations from their school (Association of American Medical Colleges 2024). These disabilities included ADHD, psychological, chronic health, learning, sensory, and mobility disabilities. Critically, accommodations have been central to research on disability in medical education to date. This is thanks to protections outlining accommodations as a legal right in the Americans with Disabilities Act, As Amended (2008). Across qualitative studies in the field, disabled medical learners have described barriers to participation beyond accommodations (administrative hurdles, stigma, lack of mentorship and institutional validation of disability), enablers to learning and clinical performance (transparent and streamlined disability services, access to mentorship and faculty champions), and reactions to ableism in medicine (fighting for a place in medicine, giving up on access) (Battalova et al. 2020; Bulk et al. 2017; Grant et al. 2019; Jain 2022; Jain et al. 2024; Shaw et al. 2023; Shrewsbury et al. 2018; Stergiopoulos et al. 2018). Within these students’ narratives there are powerful currents of feeling. These responses are laden with emotions: the fear of requesting accommodations, the hope of fulfilling implicit ideals of the ‘good doctor’, or the grief of not belonging. As researchers in the growing field of disability in medical education, we have often witnessed the emotional pull of participants’ responses, and felt our own need to understand, interpret, and respond.

In this paper, we attempt to untangle the emotional threads of disability inclusion and exclusion in medical education in a uniquely large dataset. The Association of American Medical Colleges’ Year Two Questionnaire is a national survey distributed annually to all second year U.S. medical students, and includes a free-text question on students’ experiences with disability in medical school. Our analysis of respondents’ emotions stands in parallel to a separate analysis of this same dataset, which used a critical realist approach to map the system of disability inclusion in U.S. medical education, and identify how students navigate that system (Jain et al., 2024). Given the size and richness of the dataset, the present paper analyses a separate facet of the data, this time using a sociocultural lens and constructionist paradigm to study respondents’ emotions. To do so, we propose affect theory as a way forward for studying the political character of emotions in medical education. First, we review how the study of emotions has contributed to existing medical education research. We then discuss the development and uses of affect theory outside of health professions education, including in critical disability studies. Finally, we demonstrate how engaging with affect theory allows us to study the emotions that underpin disabled learners’ subjectivities and sense of agency in an ableist system. Using emotions as our entry point, we contribute a novel understanding of how disabled medical learners perceive the possibility for systemic change.

### Emotions in medical education

Emotions are ever-present in medical education, and are central to discussions of maintaining professionalism, enhancing provider wellbeing, and ensuring patient satisfaction (McNaughton, 2013; Sukhera et al., 2025). To date, medical education research has focused on the role of emotions in learning (Artino Jr et al., 2012; McConnell & Eva, 2012; Toufan et al., 2023), simulation (LeBlanc & Posner, 2022), and the effects of emotional experiences on learners’ identities (Bynum, 2021; Bynum et al., 2021, 2019; Lönn et al., 2023). Yet the field’s engagement with emotion has been largely limited to studying emotions as universal physiological states, or as skills that can be learned, observed, and evaluated (McNaughton, 2013). In both of these frames, emotions are conceptualized as individual psychological experiences that impact learning.

These ways of studying emotion contrast with a sociocultural lens which views emotions not as physiological states or skills, but as practices that shape and are shaped by culture and society (McNaughton, 2013). Increasingly, authors like McNaughton (2013), Hirshfield and Underman (2017, 2016), Ajjawi et al. (2022), and Mills (2022) have argued that sociocultural perspectives are a much-needed path forward for studying emotions in health professions education. This sociocultural approach would allow us to study emotions beyond the level of internal psychological states by highlighting “emotion’s function in shaping social exchanges and its role in professional identity formation and practice,” (McNaughton, 2013, p. 76). It allows us to ask new questions about how emotions become expressed, taken up, and reinforced by normative conceptions of the “good doctor” in medical training.

### Contributions of affect theory

Outside of health professions education, the sociocultural study of emotions has gained critical momentum. Since the 1990s, scholars in cultural studies, literary studies, and philosophy have followed the “affective turn” to study the relations between emotions and power (Gregg & Seigworth, 2010). The resulting approach of affect theory provides a lens for studying how individually felt emotions become expressed and circulated as collective *affects.* These affects in turn align with particular sociopolitical ways of understanding the world. In this frame, affects are not synonymous with emotions. Emotions are felt individually as internal states, while affects occupy both a personal and political space. Affect theory allows us to understand the emotions we experience individually as reflections of our participation in political systems that reach well beyond individual experience. Affects work powerfully: they mediate the relationships between “the psychic and the social, and between the individual and the collective,” (Ahmed, 2004, p. 119). They align bodily spaces with social spaces, and individuals with communities. As Goodley et al. (2018, p. 199) explain, “Affects are felt individually, materially and physiologically but are always being reproduced by their entanglements in the social world.” As a result, affects can circulate outside of individuals, as the very effects of social life. The circulations of these social effects become known as *affective economies* (Ahmed, 2004).

The major currents of affect theory are paradigmatically aligned with poststructural theories of social organization and control, where affect is “as infrastructural as a factory” (Massumi, 1995, p. 106). In other words, affect provides an entry point for studying the operations and effects of power. As both an internal state and a shared social effect, affects become a key unit of analysis for investigating how individuals constitute their subjectivity and agency in a given social world. To this end, affect theory has propelled research on social injustices by highlighting the affective states that become shared across marginalized individuals. Affect theory, however, is not a uniform enterprise. Scholars have diverged in their areas of research, units of analysis, and adherence to particular disciplinary traditions (Gregg & Seigworth, 2010). We are primarily influenced by Berlant (2011) and Ahmed (2004, 2012), key thinkers who have driven the study of neoliberal, Black, and queer political subjectivities, along with feminist Foucauldian scholarship (Penz & Sauer, 2019).

The influential literary scholar Berlant (2011) developed the concept of “cruel optimism” to describe a core affect of neoliberal economies. As Berlant described, “A relation of cruel optimism exists when something you desire is actually an obstacle to your flourishing,” (p. 1). These desired objects can be concrete (food), relational (love), ideational (a “fantasy of the good life” or way of living), or political (an ideal of a political project). Berlant explains that optimistic attachments are not *inherently* cruel; however, they can become cruel when “the object that draws your attachment actively impedes the aim that brought you to it initially,” (p. 1). Berlant provides the example of increasingly precarious labour in neoliberal economies, driven by the “affective shift toward valuing lateral freedoms and creative ambitions over strict upward mobility,” (p. 193). In other words, as workers and owners favour increasingly flexible work practices to respond to market demand, this flexibility comes at the cost of increasing instability for workers amid a shrinking social welfare state. Drawing from critical theory, poststructural theory, and psychoanalysis, her goal is to understand why people remain attached to fraying fantasies of personal, political, or economic relations that ultimately harm them.

Sara Ahmed (2012) has drawn from queer, feminist, postcolonial and critical race theory to write on happiness as an affect of obedience, where “bad feelings [get] hidden, displaced, or negated under public signs of joy.” Discomfort, in contrast, can be productive by revealing normative power. Ahmed (2004) writes, “discomfort is not simply a choice or decision… but an effect of bodies inhabiting spaces that do not take or ‘extend’ their shape” (Ahmed, 2004, p. 152). Affects of anger, resistance against oppression, and refusal to “go along with it” become a form of disobedience: “to refuse the place in which you are placed, is to be seen as causing trouble.” Ahmed names the “killjoy” as the holder of negative affects, who embodies a “politics of willfulness”. Killjoys “put their bodies in the way” of human traffic and economic flow to disrupt the systems upheld, reproduced and justified by happy affects. Ahmed’s theorization helps us understand how people on the margins take up or resist oppressive ideals and practices, using affect as point of entry.

More recently, affect theory has gained traction among feminist Foucauldian scholars studying the regulation and self-regulation of emotions as an emerging neoliberal mode of social control, a phenomenon Penz and Sauer (2019, 2017) have called “affective governmentality.” Their research, which has since spurred a wave of research on gendered affective labour and its ties to state governance, has highlighted the role of affect in neoliberal power in increasingly service work-based economies (Shoshana, 2022; Warner, 2024). They argue that “affect management” of employees and public servants aims to foster an “entrepreneurial spirit” in workers and bureaucrats that emphasizes individual responsibility, while commodifying their creativity, social skills, and affectivity to make companies and governments more competitive or efficient. Their analysis focuses on affects “as bodily expressions that are always related to social and discursive contexts,” (Sauer & Penz, 2017, p. 47) and has been taken up to study how those contexts produce “self-improving, entrepreneurial subjects, responsible for their happiness and well-being,” (Smoliak et al., 2025, p. 1).

### Emotion, affect, and disability

Emotions have long been a topic of interest for disability scholars. Prior to affect theory’s introduction in the 1990s, disability scholars have highlighted the emotional nature of disability, both for disabled people and the non-disabled people they encounter. Disability often provokes anxiety and uncertainty in the non-disabled observer (Watermeyer, 2006, 2025). Paul Hunt’s (1966) early critical work on disability stigma describes his experiences with disability, stating “inevitably it produces an instinctive revulsion, has a disturbing effect” (p. 12). Critical psychoanalytic views of disability oppression theorize that this feeling of “instinctual aversion” represents “universal existential anxieties to do with the frailty, imperfection and impermanence of the body, as well as the realities of dependency and mortality,” (Watermeyer, 2025, p. 124).

Following the “affective turn”, critical disability scholars have taken up affect theory to understand the ways in which positive and negative affects work to marginalize disabled bodyminds (Goodley et al., 2018). For example, disability is culturally linked with affects of discomfort, tragedy, pity, and disgust, which produce and reinforce an ableist agenda that perceives disabled people as less-than-human. Fritsch (2013) and Kolarova (2012) have explored how positive affects like pride and hope equally function to uphold an ableist status quo. Pride becomes an act of overcoming the presumed shame of disability, while hope elicits the biomedical drive to overcome or cure disability (Fritsch, 2013; Kolarova, 2012). These positive affects position disabled people as “the inspiring crip” — the desirably disabled bodymind who can be cured, and who has individualized problems that can be known and solved (Fritsch, 2013). The myth of the “inspiring crip” or “super crip” positions disabled people as exemplary if and only if they can exceed the potential of their non-disabled counterparts. In these affective economies, regardless of valence, all roads lead to ableism. Taken together, critical disability scholars’ engagement with affect theory makes visible how emotions produce and are produced by deep-seated social ideas of disability. Within medical education research, where sociocultural perspectives on emotion are much needed, affect theory provides a unique and valuable lens for understanding the experiences of disabled students in medical education.

### Situating our orientation to disability

We operate theoretically within Kafer’s (2013) political/relational model of disability. This model refutes the biomedical model at the heart of medical education and practice, which views disability as an individual physical or mental impairment which must be treated, managed, and cured (Jain, 2022, 2024; Oliver, 1996; Reaume, 2014). In contrast, the political/relational model views disability as a result of political, social, and ideological systems that marginalize disabled people (Kafer, 2013). In this frame, disability becomes a political category, where disabled people’s experiences are shaped by their interactions with the people and institutions on which they rely. This political/relational model is central to how we conceptualize disability and its operations within medical education (Jain et al., 2024). It shapes our language choice (“disabled person” rather than “person with a disability”) as we are not referring to a first-person declaration of disability, but rather the political category they belong to by virtue of the systems they operate within and depend upon, whether they identify as disabled or not. It also aligns with our axiological commitment to improving the existing systems of disability inclusion in medical training. As Friedner (2025) writes, “access is a process, and it is a process rooted in critique and a sense that the world can and should be otherwise,” (p. 167).

## Methods

We used reflexive thematic analysis to interpret the collectively held affects circulating in texts produced by disabled medical students through a national survey. Using a combination of inductive and deductive techniques informed by affect theory and the political-relational model of disability, our study design operates within a critical paradigm using a relativist ontology and constructionist epistemology (Braun & Clarke, 2022; Brown & Dueñas, 2020).

### Dataset

The dataset comprised student responses from the 2019 and 2020 cohorts of the Association of American Medical Colleges (AAMC) Year Two Questionnaire (Y2Q), an online survey distributed to all second-year MD students across the U.S. This is the largest known national dataset reporting experiences of disabled medical students, with questions about disability first included in 2019. In total, 27,009 students participated in the survey (61.3%), and 2,438 reported having a disability (9.0%). Students reporting a disability on the survey were prompted to: “Use the space below if you would like to share anything about your experiences regarding disability and medical school.” A total of 674 disabled students responded to this question, with 336 respondents in 2019, and 338 in 2020. Responses ranged from 1 to 284 words, with a total of 31,918 words written. These free-text responses were anonymized and de-linked from the rest of the survey data by the AAMC before transferring to our team, precluding any demographic analysis of responses. Anonymization included removal of any identifiers such as institutional names, leaders, and locations. Our distinct analysis of this dataset was part of a larger study of the 2019 and 2020 AAMC Y2Q data related to disabled student experiences (Jain et al. 2024; Meeks et al. 2023; Meeks et al. 2022a, b; Moreland et al. 2024; Pereira-Lima et al. 2025). This study was approved as exempt by the University of Michigan Institutional Review Board (HUM00258874, September 2023), and was carried out in accordance with the principles of the Belmont Report (1979) and the Declaration of Helsinki.

This dataset was unique for its size, representativeness, and scope. The anonymity of the survey provided a critical opportunity for witnessing the experiences of disabled students who have been historically underrepresented in research: those who do not disclose disability to their school or seek accommodations. Previous qualitative studies on disabled medical students have primarily studied those who requested and received accommodations, which only represents approximately half of disabled medical learners (Meeks et al. 2022a). The stigma (Haque et al. 2021; Meeks and Jain 2018; Neilson 2023; Slavin 2016) and complexities of claiming (Jain 2024) disability remains a major barrier to disclosure outside of fully confidential settings offered by anonymous surveys. Thus, we aimed to highlight these voices missing from existing research, to capture a more complete picture of the disability inclusion landscape in medical education. Moreover, while individually felt emotions are often best explored qualitatively through interviews and written accounts (LaDonna et al. 2018), large-scale open-text survey responses provide unique affordances for studying affect as a collective emotional response to a shared political reality of disability in/exclusion. We developed the concept of methodological *pointillism* to describe our technique of using brief snapshots of individual experiences, compiled in large quantities through survey data, to provide a broader picture of shared social phenomena (Jain et al. 2024). By virtue of its size, the dataset allowed us to study broad affective trends, capturing brief moments of expressed emotion and situating them within a wider political and affective context. Thus, while recognizing LaDonna et al.’s (2018) caution about the use of open-text survey data for qualitative research, we argue that this dataset meets a standard for analysis in that it is “new, unique, and rare” (p. 348) and appropriate for answering a specific research question. Furthermore, we determined that the dataset had sufficient information power for the requirements of this study (Braun & Clarke 2022). Nonetheless, we acknowledge that, while a pointillist analysis offers a new and broad collective rendering of experience, further exploration of this topic through in-depth rich qualitative data (e.g., interviews, observations) would bring clearer detail to expand our understand of emotions and affect in the experience of disability in/exclusion.

This dataset underwent a first analysis by our team in 2022–2023 to map the systems for disability inclusion in medical school, and how students navigated these systems (Jain et al., 2024). This analysis remained close to the data, using semantic coding to understand systems as participants perceived them. To honour the importance and immediacy of students’ perspectives to inform efforts to dismantle ableism, we maintained a critical-realist ontology and contextualist epistemology (Jain & Stergiopoulos, 2025). Given the size and richness of this dataset, the data was generative of additional analytical approaches, paradigms, and research questions. Situating our research axiologically within efforts to advance justice for disabled people, we found that critical perspectives provided an additional layer of understanding beyond a critical-realist frame (Jain & Stergiopoulos, 2025). In our early stages of familiarization with the data, it became clear that participants’ responses were laden with emotional language. Viewing the data through the lens of affect theory positioned us within a critical paradigm to understand how power circulates in spaces of disability inclusion. Thus, the present study shifts our gaze beyond the semantic content of students’ responses to bring focus to the relations between power and emotion. In particular, we aimed to understand the affects underpinning students’ conceptions of disability, responsibility, and agency as disabled learners in ableist institutions.

### Positionality, reflexivity, and the role of researchers’ emotions

We come to this work from a variety of positions as disabled/not-currently-disabled, researchers/clinicians, and insiders/outsiders to the medical profession. We inhabit colonial geographies of the global North, as immigrants to Canada and Aotearoa/New Zealand. ES identifies as a cis White woman who works as a physician, clinical teacher, researcher of disability in medicine, and has past lived experience with chronic illness in medical training. NRJ identifies as a cis mixed woman (White and Indian) born and raised in the US who researches disability in medicine, teaches clinical education, formerly led disability resources, and lives with mental illness.

The emotions that students expressed in the data elicited emotions in us as researchers, and gave rise to rich discussion about our own emotional reflexivity vis-a-vis the data. Our reflections on our emotions thus became interpretive tools. The psychoanalytic concept of *countertransference* provides a useful parallel process to our approach. Countertransference refers to “those thoughts and feelings experienced by the [psycho]analyst that are relevant to the patient’s internal world and which may be used by the [psycho]analyst to understand the meaning of the patient’s communications,” (Bateman et al., 2022, p. 129). In this sense, we remained attuned to our own emotional responses throughout the process of coding, to gather clues about the emotional meaning of participants’ responses. As we read the data, we asked ourselves, *what am I feeling* and *what is my body feeling?* We considered our reactions in light of personal experiences, asking *is this from the student*,* or am I over-projecting my experience?* Beyond our own reactions as researchers, we also considered participants’ perceived audience of this anonymous survey—educational organization administrators—and reflected upon the emotions students intended to perform and elicit for this audience in their responses. We memoed our emotional reactions, and weaved the process of constant questioning throughout the stages of coding, analysis, team meetings, and writing. As authors implicated in various aspects and roles related to the scenarios our participants described, discussions of our own positionalities and reactions were essential.

### Analytic methodology

We used reflexive thematic analysis to analyze how affects circulated among a national collective of disabled medical students. Reflexive thematic analysis is uniquely flexible in its engagement with theory and epistemology, and is particularly well-suited to large datasets and survey data (Braun & Clarke, 2022; Terry & Hayfield, 2020). It allows the researcher to flexibly take stock of a spectrum of semantic and latent content in a dataset via both inductive and deductive coding (Braun & Clarke, 2022). Given the affordances of our dataset for generating a broader pointillist picture using “snapshots” of individual perspectives, we used a combination of deductive and inductive coding to generate both semantic and latent codes, described further below. We adopted a relativist ontology and constructionist epistemology, and grounded our analysis within the theoretical frameworks of affect theory and the political/relational model of disability.

Following Braun and Clarke’s (2022) six phases of reflexive thematic analysis, we familiarized ourselves with the complete dataset, with each author focusing on a single cohort of students. In a first wave of inductive coding, we identified individually expressed emotional meanings, along with patterns in how students oriented their relationships with their institutions. We coded direct references to emotional states (e.g., “I was really embarrassed,” “I feel guilty,” “I am grateful”) to generate *semantic codes* that captured students’ explicit meanings about their emotions. We also coded emotion-laden situations described in the data, where students did not name an emotion explicitly. These represented *latent codes*, capturing implicit or conceptual meaning about students’ emotions. For example, one student wrote: “I found the process of getting accommodations at my med school to be extremely exhausting to the point where I wished I hadn’t even tried,” which we coded as *exhaustion* and *regret*. In contexts where latently expressed emotions were less clear, we kept track of voice (active vs. passive) and tone (writing style, word choice, use of emphasis), and came to consensus on their emotional meanings in team meetings. In parallel to students’ emotions, we took stock of how they interacted with their peers, teachers, and institutions, including whether or not they sought out, avoided, declined, or felt entitled to disability support. These interactions provided insight into how students oriented themselves sociopolitically to the system and its practices. Moreover, they highlighted how these sociopolitical orientations shaped their expectations around how disabled students should be treated, managed, and included in medical education.

During this initial wave of coding, we met throughout to exchange perspectives on the two cohorts of data, during which we began to notice broader patterns between students’ emotions and the ways they related to their institutions. We saw interconnections between the types and valences of emotions students expressed (e.g. satisfaction, gratitude, dismay, regret, frustration) and the ways they perceived their sense of agency in the system and acted on seeking support or not. We termed these categories *emotion* (i.e. what students *felt*), *action* (i.e. what students *did* to seek support, protest, or laud their institution), and *responsibility* (i.e. how students *responsibilized* their disability as either personal or systemic, and who or what was *responsible* for changing). We developed a framework for linking these categories and locating the patterns among them. We conducted a second deductive wave of coding to explore how this framework fit the data, identifying moments when students expressed a particular emotion in conjunction with their reported actions and/or sense of responsibility. For example, one student wrote, “I am honestly amazed at how supportive the administration and deans office has been to me… For their willingness to help, I am very grateful for the administrators for… helping me face the barriers that were hindering me from reaching my full potential.” The student’s sense of amazement and gratitude (emotion) was linked with seeking and receiving institutional support (action) for a disability which they perceived as their own responsibility to manage (responsibility).

The work of clustering specific patterns of *emotion*, *action*, and *responsibility* allowed us to generate three dominant *affects*, highlighting three diverging affective ways of being and knowing disability in U.S. medical education. We returned to the data to develop detailed analyses of these three affects, drawing from critical disability theory and affect theory to inform our interpretations. Affect theory allowed us to make sense of the relationships between disabled students’ emotions and their negotiations of their institutions’ responses. It allowed us to interpret affects as vehicles for institutional compliance versus sites of resistance. Critical disability studies provided an additional layer by contextualizing those emotional negotiations against a background of disability in/justice. In so doing, we tracked patterns in students’ alignments with particular notions of disability (biomedical or social) and with social groups (with or versus the institution), and how these related to their emotions. To be clear, we used the designation “social” as a heuristic to categorize statements that indicated an understanding that non-internal or non-biomedical forces have a role in creating disability.

## Affective economies of ableism in medical education

Students related dozens of individually expressed emotions in their responses, including amazement, anger, annoyance, awe, bitterness, contentedness, disappointment, discouragement, disgust, dismay, dissatisfaction, exasperation, exhaustion, fear, frustration, gratitude, heartbreak, hope, hopelessness, hurt, indignation, overwhelm, pleasure, regret, resignation, righteousness, self consciousness, shame, shock, surprise, worry, and unhappiness. As we took stock of the sheer number and magnitude of emotions expressed, we noted three major polarizations throughout the dataset. These three poles represented: (1) students who were happy with the system, (2) students who were frustrated by the system, and (3) students who were resigned to an ableist status quo.

**Fig. 1:**
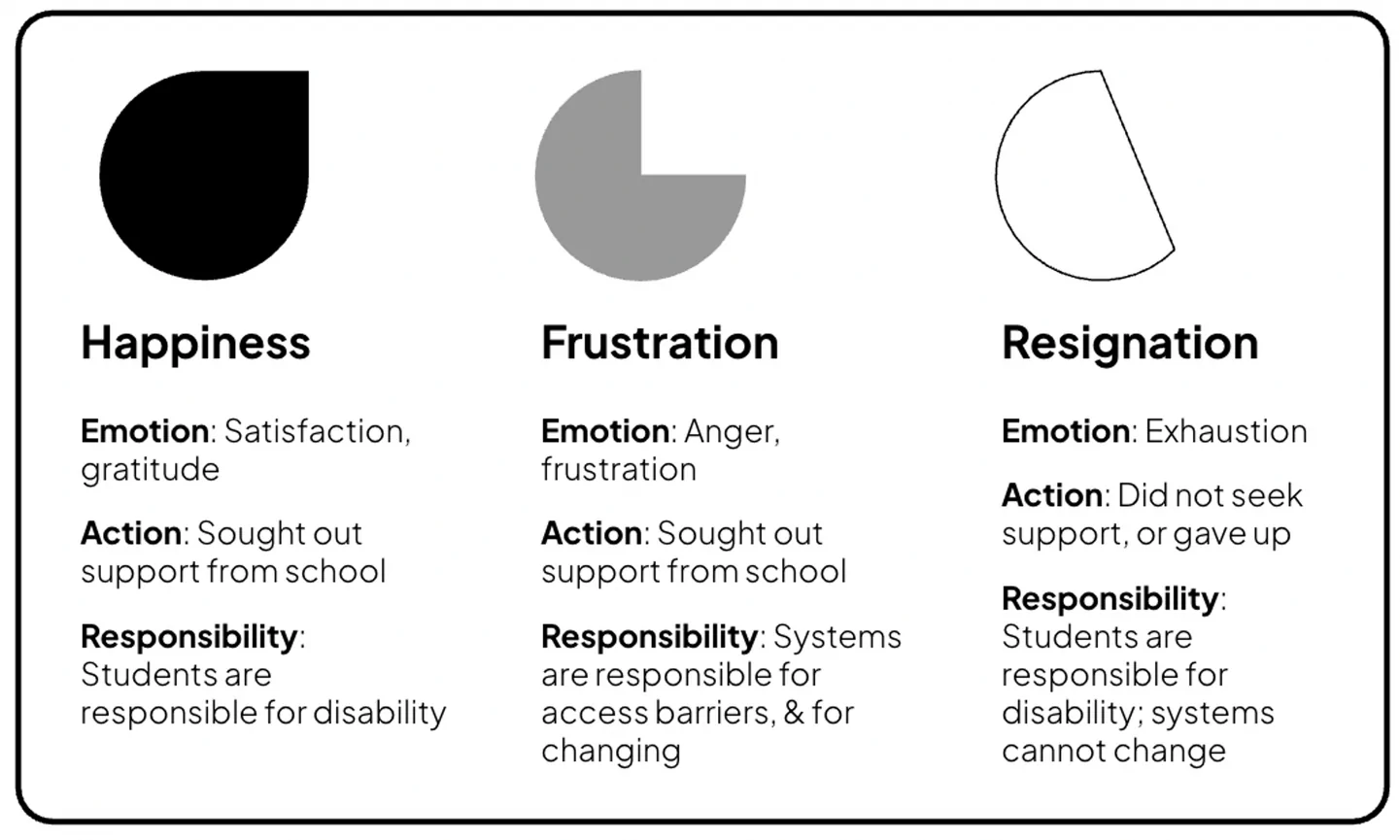
Clusters of affects (emotion, action, responsibility) in responses of disabled U.S. medical students

These initial emotional poles mapped onto three major clusters of emotion, action, and responsibility that we tracked across student responses. We termed these clusters *affects*, for their ability to capture individual and collective emotion alongside effects of power. We analysed each combination of emotion, action, and responsibility to understand broader collective affects that resulted from students’ social realities. Affect therefore provided an entry point for understanding and critiquing the institutions, relationships, and systems that gave rise to the feelings students expressed. Figure 1 summarizes this clustering of affects, and depicts what students expected in relation to what they received: happy students expected little and what they received went beyond their expectations; frustrated students expected compliance with disability law and found the system lacking; resigned students cut off their expectations altogether.

The affect of happiness circulated in accounts of students who had reached out for help (action), which they found satisfying (emotion), and where they often implicitly or explicitly deemed their disability a personal responsibility (responsibility). The affect of frustration (emotion) ran through accounts of students who had tried to seek support (action) which fell short, and who held their schools responsible for learning barriers and for improving (responsibility). Resignation was the third affect, in which students described giving up on access (action), and internalizing the responsibility for their disability in a system they did not feel they could change (responsibility), leaving many students feeling worn out (emotion).

Student responses did not always fit neatly into specific affects. Some students expressed themselves with multiple affects in response to different institutional experiences, while others blended particular emotions with non-concordant actions or agency (e.g., a frustrated student who did not act to seek support). We consider these affects as clusters rather than categories, to account for the variations we saw across students’ experiences. In this sense, they mirror the use of *ideal types* used in sociological research, which provide a heuristic device for sorting empirical data rather than delineating bounded categories (Crotty, 1998; Weber, 1978[1922]). Table 1 provides an example of this clustering. In the sections that follow, we detail our analysis of each affect.

**Table 1:** Examples of emotion-action-responsibility framework

Student response	Emotions, actions, responsibility	Affect
“At my med school, you have to sign up to get clinical experience, such a suturing, putting on casts, etc. The spots fill up so fast and if you’re already overwhelmed with the burdens of school, it’s hard to sign up in time for the awesome hands-on activities. I wish the faculty would be more supportive and instead add those elements to the curriculum. It is really hard to be able to focus on multiple things and have to do so many things on my own. I am not sure if this is too much to ask for because I see a lot of my classmates succeeding, but I feel so overwhelmed.”	**Emotion**: Overwhelm	Resignation
	**Action**: No explicit action taken; giving up	
	**Responsibility**: Personal responsibility to “keep up”, system responsibility to provide support	
“NBME treats us like we don’t matter and the process to get accommodations makes us feel that you don’t believe that we have a disability. It’s disgusting.”	**Emotion**: Disgust	Frustration
	**Action**: Applying for accommodations in long and taxing process	
	**Responsibility**: System responsibility to provide disability support	
“I am honestly amazed at how supportive the administration and dean’s office has been to me. They really make sure that I am connected to every single form of support and resource to help with my disabilities.”	**Emotion**: Amazed	Happiness
	**Action**: Applying for disability resources from multiple responsive administrators	
	**Responsibility**: Implied student’s responsibility to reach out and access existing support for “my” disability	

### Happiness

The affect of happiness circulated among students who had reached out to their institutions for support, received it, and were satisfied. They did not express a need to change existing institutional arrangements; they were content with the status quo. Students described feeling “pleased”, “amazed”, “proud”, “satisfied”, “appreciative”, “incredibly impressed”, and “grateful.” They detailed interactions in which their school “promptly helped me”, “was very efficient”, “was extremely helpful and attentive”, “has been very supportive”, “has done a tremendous job”, or “has been amazing about accommodating me!” They demonstrated unwavering trust in their institutions, with one student stating, “If I had a request, I’m 100% sure the administration would help me in any way that they could.” Interestingly, this student noted earlier in their response that they had never actually made a request.

Across responses, happy students tended to frame their disability as a personal rather systemic responsibility. Disability was an individual problem, to be solved through the student’s own self-management and, in some cases, through medical treatment. Students mentioned their diagnoses and symptoms, or described explicitly personal concerns related to disability which they called “my issues” or “my struggles”. They detailed various aspects of their lives such as diet, exercise, and sleep, which they used to “support management of my chronic illness”, emphasizing their expectation of self-management. They rarely referred to functional limitations related to barriers in their learning environments. In this framing, students implied that they did not expect their schools to provide additional support for their learning. This expectation came across in responses where students felt “endlessly grateful” for any institutional support they received, positioning this support as exceptional and rare. Many students described their schools going “above and beyond” to “meet [their] needs” as disabled learners. As one student wrote:

*My medical school has gone above and beyond to accommodate my schedule in the midst of absences for appointments, procedures, and exacerbation of symptoms. I feel incredibly grateful for the constant support from faculty, staff, and student affairs regarding my situation.*

Here the student frames the school’s provision of legally mandated accommodations as going “above and beyond”. In turn, compliance with disability law is transformed into an act of institutional generosity for which the student is “incredibly grateful”. Disability is relegated to “my situation”; it is the student’s problem, framed here as fortunate for receiving institutional benefaction.

Other expressions of happiness were even more emphatic, pronouncing a sense of awe at the very possibility of accommodations in medical training. In these responses, students described disability services or administrators as “excellent”, “absolutely fantastic,” “phenomenal”, “truly incredible”, “more than helpful”, “overwhelmingly helpful”, “INCREDIBLY helpful”, “extraordinarily empathic”, and “truly my hero.” One student wrote that their positive experiences came from a sense that “the school genuinely cares not only about my ability to perform despite my condition but also about my overall wellbeing.” Embedded in this positive experience, however, was the admission of the student’s actual expectation: that medical education only cares about students’ performance, and that disability (not learning barriers) gets in the way of performing. Another happy student wrote:

*I have been EXTREMELY pleased with [my school]’s handling of all aspects of my mental health needs. Student mental health services is wonderful, accessible, and non-judgmental. Many faculty and staff are public and vocal about having experiences of acute and chronic mental illness and have organized events to talk about mental illness in the field of medicine. That climate and those events have truly minimized the stigma that I experience as a student and a future doctor with chronic mental illness.… I have found that [my school] acknowledges that such experiences among medical professionals are not detriments or things to hide but instead that they ADD WORTH to our work and help the field as a whole work more effectively and empathetically with patients.*

These positive experiences are heartening. They present a social reality in which disability inclusion is possible and even welcomed in medical education. Yet as we immersed ourselves in the data, we sometimes felt in ourselves an undercurrent of disappointment. Amid these expressions of joy, students often appeared to be begging for crumbs. Even basic and unquestionable measures of access were positioned as exceptional gifts to students. For example, one student with diabetes wrote, “They have been very accommodating of letting me bring my insulin pump and food to all activities, including tests.”

Lauren Berlant’s (2011) concept of “cruel optimism” brings nuance to affects that appear “happy” on the surface. As Berlant writes, “an optimistic attachment is cruel when the object/scene of desire is itself an obstacle to fulfilling the very wants that bring people to it,” (2011, p. 227). The same can be said for medical education, which has increasingly opened its doors to diverse (including disabled) students. While the trend toward increasing diversity may inspire optimism, it becomes cruel when the very diversity these students were selected for renders them unable to participate, particularly where educational environments remain inflexible to their needs. In happy students’ responses, we noted an underlying discomfort, which circulated most clearly through moments where students appreciated the accommodations their institutions made available, but regardless, chose not to seek them out. In this context, discomfort was the effect of disabled bodyminds existing in spaces in which disability was never meant to belong (Ahmed, 2004). As one student described, “One works so hard to get into medical school and give the impression of profound capability. Thus, I would feel uncomfortable requesting any accommodations.” Disability and the need for accommodations signalled a state of poor fit in medical education, resulting in the social effect of students’ discomfort.

In other settings, schools’ efforts at inclusion were described as good, but not good enough. These accounts collapsed the idealized image of their schools’ inclusion efforts, and made students’ discomfort more explicit. These students strayed from the ideal type of the happy affect, expressing satisfaction with the status quo on some matters, while expecting and demanding better in other aspects of their education. One student wrote:

*[The medical school] has an excellent disability services coordinator who goes above and beyond to make sure [the school] is both compliant and welcoming to students with mobility concerns. I would like to see the institution make the main campus building more accessible. Many features are outdated. Why are the handicapped parking spots only located next to a set two heavy manual doors? Please invest in better building compliance. I recognize that [the medical school] has many financial constraints*,* but this is pretty egregious. We can and should be doing better for our community.*

### Frustration

The affect of frustration ran in sharp contrast to the happy student. Frustration circulated in accounts of students who had sought support from their schools, only to have their requests silenced, ignored, partially unmet, or denied. In response, students described actively resisting institutional policies, language, and everyday practices to call out their ableism. They described feeling “frustrated”, “extremely sad”, “discouraged”, and “disappointed”, with implicit references to feeling angry, bitter, and exasperated by their experiences of exclusion. They described the institutional behaviours they’d encountered as “disgusting”, “flagrant”, “inappropriate”, “ridiculous”, “abusive”, “prejudiced”, “extremely ableist”, “absurd”, and “laughably behind.” One student noted, “It seems as if they want to discourage us from even applying [for accommodations].” These students expressed an overall distrust of their institutions, along with a need to clearly express their dissatisfaction and to self-advocate. As two students wrote:

*I have not been satisfied with accommodations. I have had to do a lot of self-advocating, and I frequently feel as if I were a nuisance. There is a lack of creativity and innovation in accommodating students at [my school], which is very frustrating since a considerable amount of marketing goes into the message that it is inclusive, innovative, accepting and radical. The disconnect between marketing and reality has been an incredible source of frustration for many students, even so far as to cause several to leave.*

*I went to my advisor to ask what kind of help I could receive because I was going through a particularly difficult flare with my condition. She literally had no suggestions except for “you can take a medical leave” and pushed me to take a medical leave. I was so frustrated because that’s not what I came for or what I was asking for. I was simply asking for help with what kind of academic resources were available for help.*

Other students used their frustration to demand more from their schools. For example, one student’s account described confidentiality breaches that jeopardized their training and career prospects: “[The school] did all of this with a cavalier attitude of ‘this is just how it is.’ I say - this is not good enough. It is time to shape up.” To extend Ahmed’s (2012) concept, these accounts inhabited the position of the *disabled killjoy*. In this position, discomfort is not in shadow, but named out loud. Everyone, including the non-disabled, is made to feel uncomfortable. Disabled killjoys agitated, expressed dissatisfaction, and asserted their rights. In contrast to the gratitude of happy students, who appeared in awe of any institutional support they received, disabled killjoys did not hesitate to call out where institutions fell short. They demanded inclusion, explicitly naming that they do “deserve” support, and that “people w[ith] disabilities [have a] right to belong in medicine.” One student wrote:

*Disability is so often discussed in ways that are so harmful by our professors. I feel like I spend a ridiculous amount of energy teaching and correcting harmful views of disability that I am left with little energy for learning. In addition, because we see SO many different professors, I am constantly having to advocate for my accommodations to be implemented, or even for a seat when I’m in clinic. The level of ableism in medicine is absurd. It’s been so hard to build a healthy social life as well, given how many assumptions are made about me as a disabled person. Finally, I can’t even use the same door as my fellow classmates due to the inaccessibility of the physical building.*

In these responses, students tended to frame their disability as produced by the system, rather than a personal problem solvable via self-management. They held institutions responsible for creating appropriate and accessible learning environments, which they identified as the source of the learning barriers they experienced. Students thus referenced functional limitations and specific barriers they faced at their schools, rather than focusing on their symptoms and how they managed them. They viewed disability as produced by rigid and restrictive curricula that failed to meet their learning needs. In this sense, failure belonged to the system, not the student. As one student described, “the school has failed me overall to make receiving accommodations [outside of test settings] possible.” In many accounts, identifying barriers became an impetus for change:

*Our lecture style set up is not always conducive to my learning style. I rarely if ever use our school’s educational resources, and anytime I am expected to attend lectures when they are mandatory, it holds me back from actually learning. I use outside sources that are more ADHD friendly, but sometimes it feels like I am penalized for not using our school’s resources (requiring attendance, not getting grade points for questions asked in class).*

Not all frustrated students saw their schools as responsible for accommodation, and not all signalled the need for change. One student described the “weirdly complicated” process of seeking out neuropsychological testing for ADHD, which resulted in a number of dead ends and unnecessary appointments: “I don’t quite know what to think about the experience. I don’t think it’s my school’s job to hold my hand through this. It’s just been a bit frustrating.” While these students witnessed and called out system failures, they deviated from the “ideal type” of the frustrated student who placed responsibility on systems, demonstrating some variability in the affects that circulated across the data.

### Resignation

The affect of resignation circulated through accounts of students who had given up on inclusion altogether. These were students who had requested accommodations that ultimately came up short, or who chose not to request them in the first place. They did not expect the system to change, and did not agitate for a better future. Students expressed feeling “exhausted”, “overwhelmed”, “unsure”, “concerned”, “worried”, “regretful”, “disheartened”, “heartbroken”, and “self-conscious.” Compared to happy or frustrated students, who often foregrounded their interactions with specific people or their schools, resigned students tended to limit their accounts to personal experiences that they faced in isolation. Disability was experienced alone. As one student wrote, “It is really hard to be able to focus on multiple things and have to do so many things on my own.” These students overwhelmingly described that “it is hard”, “difficult”, “challenging”, “a struggle”, or “when the cards are stacked against you, it’s hard to win.” In the face of these difficulties, their solution was simply to adjust, even when they saw disability as produced by the system. As one student wrote:

*I never considered my ADHD to be a disability until medical school. I just saw my brain as different. Medical school is bringing out the challenges I have with ADHD front and center with myself. It is a constant struggle, and I am constantly trying to adapt and adjust.*

Many students described their need to adapt to medical education, which they perceived as an inevitable reality. They described their disabilities as “just something I have to deal with”, or “just something to learn to live with unfortunately.” Students variably located the responsibility for disability in the school or in themselves, with some students pointing to barriers and functional limitations alongside their symptoms. However, across responses, students did not expect the school to be accountable for changing. Barriers were a given, and therefore students absorbed the burdens of self-management and surplus effort themselves. This resulted in expressions of profound fatigue, with one student writing, “I feel my physical and emotional body becoming exhausted by the hours and energy required by medical education.” Another wrote, “I stopped advocating for [my accommodations] to be enforced because it took up too much of my time and energy.” As another student described:

*My seizures are provoked by sleeping less than 8 h per day and chronic stress. I feel this condition makes my tenure at medical school and future life as a physician rather difficult. I have to care for myself and prioritize sleep above medical school, and this has always burdened me with falling behind, not learning material, and not performing well in class.*

Many statements began with “I wish.” Yet students did not seem to believe these wishes could ever come true. They expressed that, “I wish it were not so difficult”, “I wish there was more support for students with various mental illnesses”, “I wish there was more support with applications for licensing exam accommodations or workplace accommodations”, “I wish the faculty would be more supportive”, “I wish medical students were more open and understanding of psychological disabilities”, “I wish my school can do more than provide accommodations during tests”, “I wish that there was more financial support offered for students with disabilities”, “I wish medical education overall were more forgiving”, “I wish there was someone I could tell about this or someone I could ask advice.” In each response, the wish was perceived as unfulfillable based on system constraints—such as resources, stigma, or a lack of institutional desire. Some students explicitly stated their feared outcome if they actually asked for what they wished for: “I don’t want to get kicked out or ruin my career.” Students thus defaulted to not asking in the first place. As one student wrote:

*I wish the steps were more streamline[d] and there were actual protocols in place for accommodations. Even though I have specific accommodations*,* implementation seems rushed or put together last min[ute]. Sometimes it seems easier to not use my accommodations because it’s too inconvenient for staff to implement.*

In the face of these barriers, some students questioned their belonging in medicine, interpreting their lack of access as a personal failure. One student wrote, “I worry I am already burnt out and that medical training is not meant for someone like me.” Another wrote:

*I often feel like I will be a bad doctor because of my learning disability and traumatic experiences. I have faced a lot of academic difficulty and that makes me feel like a failure*.

A subset of resigned students took on a position of rugged individualism. These students acknowledged difficulties, and at the same time used the language of overcoming, surmounting, and grinding through their training. Their responses lacked explicit references to emotion, and latent emotions were largely “neutral”. They typified a bootstrap mentality, placing the entire burden of access on themselves and perceiving complaint as a form of weakness. The assumption that the system could not change was a constant; however, these students did not express a “wish” for more. They took pride in overcoming a system stacked against them, doing this entirely on their own. As one student wrote:

*I have trouble focusing, but I’ve learned to overcome that on my own. I don’t expect the school to assist me in this; it’s a part of being an adult*.

All resigned students, from the rugged individualists to the defeated wishful thinkers, demonstrated an adherence to ideals of self-regulation (i.e., regulating one’s behaviour in accordance with dominant norms). “Doing it on my own” was the only option in the face of a system they perceived as unchanging. Self-regulation also extended to the emotional sphere, with students regulating both their behaviours and their emotions in various ways. Rugged individualists’ responses were often emotionally “neutral”, not expressing a discernible emotion even in their responses to an anonymized survey. They described challenges, but the feelings behind them had been snuffed out. Other resigned students described their worries and exhaustion in the survey privately, through wishes they never expected to come true, but did not express them out loud to their schools. Penz and Shauer’s (2019) concept of affective governmentality captures the ways in which taken-for-granted ideals of emotional regulation work to produce the neoliberal subject. These subjects are individualized and responsibilized: they freely take up ideals of self-regulation as self-evident, ensuring they conduct themselves according to this ideal. As one student expressed, *“*I do not want to be regarded as or ‘labelled’ as ‘less than’. so I have not succumbed to seeking out help.”

## Concluding discussion

Emotions are inherently political. While health professions education has long treated emotions as individual psychological states, studying affect allows us to understand how emotions are reflective of broader collective relationships with power. Our study foregrounded disabled medical students’ emotions to demonstrate that how students *feel* is intimately linked with how they *act* to resist, accept, or reinforce an ableist status quo. In U.S. medical education, happy students were those who asked for support, received it, felt they belonged, and felt content with the system as it existed. Students expressing frustration were those whose requests for support went unmet, and who refused to adapt to a flawed system, advocating instead for system change. Students expressing resignation similarly perceived problems in existing disability inclusion efforts, but believed institutional change was simply impossible: it was up to them to adapt or leave medicine entirely. Underpinning these affects was an ableist culture of medicine where disabled students frequently faced scrutiny, experienced unmet support needs, feared downstream professional consequences, and even questioned their belonging in medicine. Taken together, these experiences revealed an ableist system in which disability was devalued and considered sub-optimal for the practice of medicine.

How, then, should educators respond to students’ emotions? We recommend caution and nuance to administrators and clinical faculty when interpreting students’ feelings about disability inclusion. In turn, we invite educators to notice and remain curious about the emotions we feel in response to students, and the social conditions that produce those feelings.

Having happy students, for example, may be heartening for educators; but it does not mean the system is working at its best. Many students in our sample expressed extreme gratitude at receiving the bare minimum of access, reflecting how low their expectations for institutional support were to begin with. These low expectations existed for a reason: students had learned that access was not guaranteed. While one student in our sample was pleased to be allowed to bring their insulin pump to tests—a considerably low bar—another wrote that wearing their insulin pump was forbidden in exams, incurring potential serious harms on the student’s health and performance. For educators, grateful students with low expectations for access might be considered “low-maintenance,” and even “a pleasure to work with.” They do not create friction, and allow systems to carry on as designed. However, we must consider whether expressions of satisfaction are actually signs of obedience in settings where students perceive no other option but to comply. As Ahmed (2012) writes, in these settings, “bad feelings [get] hidden, displaced, or negated under public signs of joy.” We must remain vigilant to ensure inclusion efforts do not simply replicate the existing mode of cruel optimism for disabled students, where disability is tolerated in medical education, but not actually welcomed.

In contrast, frustrated students may elicit discomfort in educators. Far from being frictionless, they throw a wrench into the works by pointing to where systems fail. As individuals they might be labelled in back-room conversations as “difficult” or “entitled.” Yet if we consider frustration as a collective affect extending beyond individuals, we saw this position as a politicized refusal to adapt to an inequitable system. In our sample, frustrated students were not asking for much: they were advocating for basic mobility access, implementation of approved accommodations, or minor flexibility in curricula and schedules. Their frustration stemmed from constantly needing to self-advocate for the minimum of legal compliance with disability law, representing potentially costly act of resistance (Jarus et al. 2023b; Wyatt et al. 2024). When students become skilled advocates, schools may come to rely on them to drive inclusion efforts and become volunteer voices of change. Yet we must also consider schools’ responsibility for improving access: students cannot be the only ones tasked with the work of change in a space where their primary task is, first and foremost, to learn the work of medicine.

Finally, schools receiving few to no accommodation requests may believe they have fewer disabled students enrolled in their program, or that disabled students receive adequate program access under current conditions. However, our analysis suggests these students may have simply given up. Many students in our sample were resigned to the system as it was, feeling so worn down they stopped asking for support altogether, while privately wishing for more. They relied on self-regulation to navigate their education, while questioning their belonging in the profession. Although fewer accommodation requests might come as a relief to educators in already-strained systems, it might also signal that pathways to support are too complicated or effortful for students to navigate (Jain et al., 2024), making students forego support altogether.

Our findings also shed new light on old questions. Existing literature on disabled medical students has frequently hypothesized that students hesitate to disclose disability to their schools due to stigma (Jain 2024; Jarus et al. 2023a; Meeks et al. 2022a; Stergiopoulos et al. 2020). However, the many ‘rugged individualist’ students in our sample demonstrated that non-disclosure was not always a matter of stigma. Many students internalized the standard of self-regulation as a sign of the “good doctor”, which was codified into policy and downstream practice through institutional warnings that accommodations were not possible in residency or in practice. Our results add to the literature on disability non-disclosure, complicating our understanding of this phenomenon, and revealing the cultural assumptions of medical training at its heart. For those students who felt they needed but did not receive accommodations — either due to non-disclosure or having requests denied or unimplemented — our findings also add to the literature on academic and personal consequences of managing medical training without disability access (Eidtson et al. 2025; Meeks et al. 2022a, b).

This analysis extends previous work in critical disability studies and medical education. As critical disability scholars have argued, positive and negative affects can work in parallel to marginalize disabled bodyminds (Fritsch, 2013; Goodley et al., 2018). In our study, happiness, frustration, and resignation represented vastly different affective positions; yet they were all responses to the same system, which rewarded low expectations and silence, while simultaneously groaning under the demand for change. Understanding these institutional responses within the specific context of medical education, the affective economies of happiness, frustration, and resignation reveal implicit expectations about what makes a “good doctor,” and reinforce what Jain (2022) has termed the *capability imperative* of medical training, whereby doctors and learners are expected to be selfless superhumans who must be malleable enough to meet extraordinary demands. Students’ accounts of giving up on access resonated with the growing work on marginalized students’ sense of (non-)belonging in medical education (Breheny et al., 2025; Bullock et al., 2024; Butler et al., 2019; Pride et al., 2024; Wyatt et al., 2020), while the imperative toward self-regulation echoed contemporary discourses of professionalism that prioritize self-management over system change to achieve wellbeing and optimal performance (Aubrecht, 2012; Stergiopoulos et al., 2018).

This study had limitations and affordances. While analyzing a large national survey allowed us to produce a pointillist picture of a vast system, we were unable to probe more deeply into students’ expressions of emotion as we would in interview-based research. Moreover, given the survey’s confidentiality requirements, we were unable to link individual responses to demographic markers (e.g., gender, race/ethnicity) to theorize impacts of intersectionality on students’ experiences of ableism. Future research could benefit from an explicit intersectional analysis of emotion and affect. Our dataset only captures the experiences of those respondents for whom “disability” is a legible identifier for their experiences, and who felt safe to share their perspectives in this survey (Jain, 2024). Notwithstanding, the confidential nature of the survey allowed for incredibly rich responses from a population which has historically avoided disclosure and remains underrepresented in research. Furthermore, while we did not ascertain a distinct affective pattern in the 2020 dataset cohort relative to 2019, we acknowledge that its specific temporal origins (i.e., amid a global pandemic) may render this data unique relative to other time periods. Using affect theory allowed us to make sense of individually expressed emotions at a broad scale that went beyond the specifics of particular schools. These snapshots of individual emotional experience allowed us to analyse affects as collective experiences produced in a shared system.

It is time for new tools for researching emotions in medical education. Studying affect provides a much-needed sociocultural lens on emotion, and offers a novel way of understanding disability inclusion in medical education. Ultimately, how students make sense of their emotions, actions, and responsibilities influences what they perceive as doable, sayable, and imaginable in the world of medical training and practice. With our minds attuned to affect, we can begin to extend beyond existing systems’ limited picture of inclusion, and imagine how true access in medical education can take shape.

## Data Availability

No datasets were generated or analysed during the current study.
